# Unique type of isolated cardiac valvular amyloidosis

**DOI:** 10.1186/1749-8090-1-38

**Published:** 2006-10-25

**Authors:** Shehzad Iqbal, Salma Reehana, David Lawrence

**Affiliations:** 1 Cardiothoracic Surgery, Heart Hospital, University College of London, London, UK

## Abstract

**Background:**

Amyloid deposition in heart is a common occurrence in systemic amyloidosis. But localised valvular amyloid deposits are very uncommon. It was only in 1922 that the cases of valvular amyloidosis were reported. Then in 1980, Goffin et al reported another type of valvular amyloidosis, which he called the dystrophic valvular amyloidosis. We report a case of aortic valve amyloidosis which is different from the yet described valvular amyloidosis.

**Case presentation:**

A 72 years old gentleman underwent urgent aortic valve replacement. Intraoperatively, a lesion was found attached to the inferior surface of his bicuspid aortic valve.

Histopathology examination of the valve revealed that the lesion contained amyloid deposits, identified as AL amyloidosis. The serum amyloid A protein (SAP) scan was normal and showed no evidence of systemic amyloidosis. The ECG and echocardiogram were not consistent with cardiac amyloidosis.

**Conclusion:**

Two major types of cardiac amyloidosis have been described in literature: primary-myelomatous type (occurs with systemic amyolidosis), and senile type(s). Recently, a localised cardiac dystrophic valvular amyloidosis has been described. In all previously reported cases, there was a strong association of localised valvular amyloidosis with calcific deposits.

Ours is a unique case which differs from the previously reported cases of localised valvular amyloidosis. In this case, the lesion was not associated with any scar tissue. Also there was no calcific deposit found. This may well be a yet unknown type of isolated valvular amyloidosis.

## Background

Amyloid deposition in heart is a common occurrence in systemic amyloidosis. But localised valvular amyloid deposits are very uncommon. It was only in 1922 that the cases of valvular amyloidosis were reported [1]. Then in 1980, Goffin et al reported another type of valvular amyloidosis, which he called the 'dystrophic valvular amyloidosis'. We report a case of aortic valve amyloidosis which is different from the previously described isolated valvular amyloidosis.

## Case presentation

A 72 year old gentleman was admitted with shortness of breath and palpitations via accident & emergency department. He was found to be in atrial fibrillation and in left ventricular failure. He was a hypertensive diabetic with poorly controlled blood sugar. Examination revealed an ejection systolic murmur. His left ventricular failure was treated with diuretics. Transthoracic echocardiography showed a stenotic bicuspid aortic valve, with a valve area of 0.6 sq.cm. and a peak gradient at rest of 90 mm Hg. His pulmonary artery systolic pressure was 50 mm Hg. A lesion attached to the inferior surface of the leaflets and extending onto the basal septum was visualised.

He was referred for urgent aortic valve replacement following stabilisation with diuretics. At operation, the lesion was found attached to the inferior surface of his bicuspid aortic valve. It abutted but was not attached to his ventricular septum. The valve and the lesion were excised intact and the valve replaced with a 23 mm mechanical prosthesis.

The patient had a prolonged post-operative phase complicated by respiratory infection. However, he went on to make an excellent recovery and was discharged home.

## Results

Histopathology examination of the valve revealed that the lesion contained abundant amorphous eosinophilic material. The presence of amyloid deposits was demonstrated by positive staining with congo red and with apple green birefringence. Staining was performed using monospecific antibodies reactive with serum amyloid A protein (SAA), apolipoprotein (apoAl), transthyretin (TTR) and with kappa and lambda immunoglobulin light chains. These investigations were negative, concluding that this was a non-AA type deposit and AL (monoclonal immunoglobin light chain) amyloidosis was the most likely diagnosis.

Further investigations and follow up were carried out in the Centre for Amyloidosis and Acute Phase Proteins, NHS National Amyloidosis Centre in the Royal Free Hospital London. Review of the valve histology confirmed the presence of abundant, often nodular amyloid deposits within a fibrinous mass; the amyloid did not stain for kappa or lambda, SAA, Apolipoprotein AI or TTR. Sequencing of TTR gene was wild type.

The serum amyloid A protein (SAP) scan was normal and showed *no evidence of systemic amyloidosis *[2]. The ECG and echo were not consistent with cardiac amyloidosis. There was no evidence of paraprotein in serum or urine. The serum free light chain assay was normal. Serum creatinine was 78 ml/min, measured clearance 82.8 ml/min, 24-hour urine protein loss 0.3 g and serum albumin was 47 g/l. Remaining biochemistry and full blood count was normal.

## Discussion

There are two major types of cardiac amyloidosis described in literature: the primary-myelomatous type, which occurs in association with systemic amyolidosis, and the senile type(s), in which the heart is involved in a more localised fashion [4]. Recently, a third type of cardiac amyloidosis, which restricts to the heart valves, has been described, first by Goffin and then by Falk et al. This localised valvular amyloidosis has been named 'dystrophic valvular amyloidosis' [3,4]. It was noted that the valvular amyloid deposits were more frequent in mitral and tricuspid valves than in the aortic valve [3].

In most of these reported cases, the valvular amyloid deposits were associated with some scar tissue which was presumably the result of some chronic mechanical trauma [5] or inflammatory process [3].

Also, in all of the previously reported cases, there was found to be a very strong association of localised valvular amyloidosis with calcified deposits. In a study by John H. Cooper et al [4] which encompassed 152 surgically resected heart valves, all amyloidotic heart valves showed some degree of calcification, and conversely 81 percent of calcified valves showed amyloidosis. The same association between amyloidosis and calcification was confirmed in the study by Falk et al [5], which showed that amyloid deposits were found in all of 39 severely calcified aortic valves.

## Conclusion

Ours is the second only reported case (after Groves et al [6]) which differs from the previously reported cases and studies of localised valvular amyloidosis.

In this case, the lesion on the aortic valve was not associated with any scar tissue. Also there was no calcific deposit found on histopathology examination. Either this was a yet unknown type of isolated valvular amyloidosis or a variant of the already described dystrophic valvular amyloidosis, is unclear.

**Figure 1:**
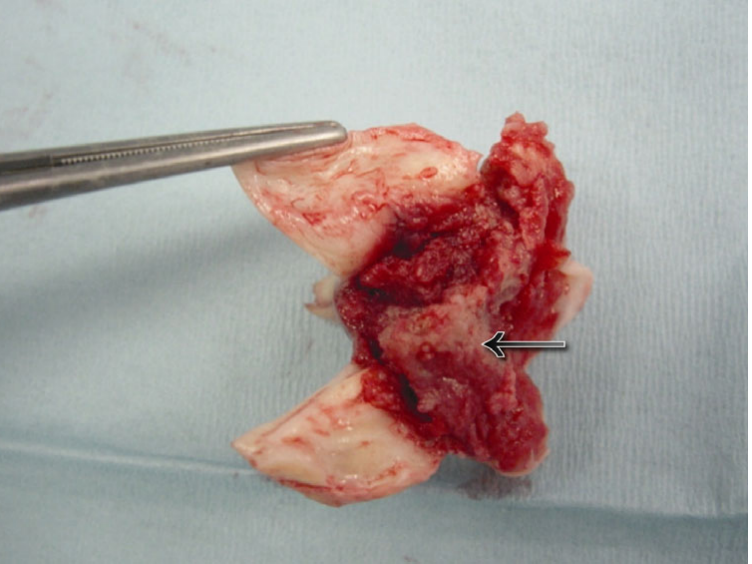
Lesion on the inferior surface of bicuspid aortic valve.

**Figure 2:**
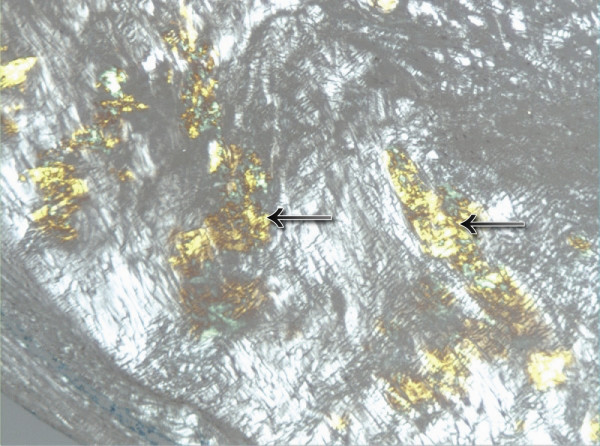
Amyloid deposit in aortic valve lesion, stained with apple green birefringence

**Figure 3:**
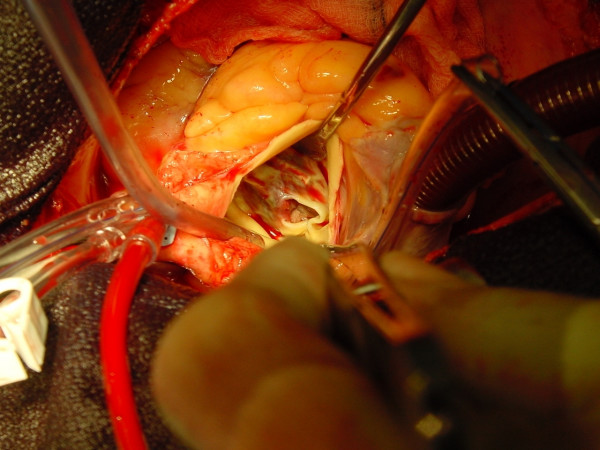
Intra-operative picture.

**Figure 4:**
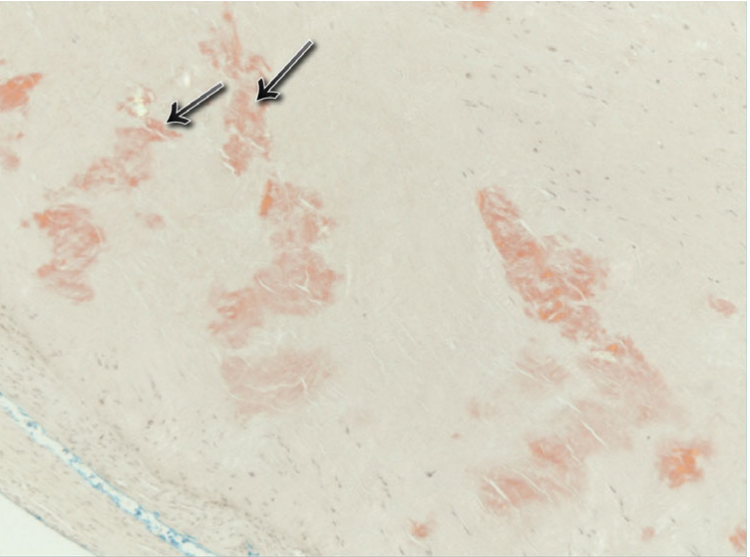
Aortic valve amyloid deposit, stained with congo red.
